# Patient Satisfaction in the Peruvian Health Services: Validation and Application of the HEALTHQUAL Scale

**DOI:** 10.3390/ijerph17145111

**Published:** 2020-07-15

**Authors:** Fernando Barrios-Ipenza, Arturo Calvo-Mora, Félix Velicia-Martín, Fernando Criado-García, Antonio Leal-Millán

**Affiliations:** 1 Escuela de Posgrado, Universidad Continental, 15046 Lima, Peru; 2 Departamento de Administración de Empresas y Marketing, Universidad de Sevilla, 41018 Sevilla, Spain; schmidt@us.es (A.C.-M.); velicia@us.es (F.V.-M.); fcriado@us.es (F.C.-G.)

**Keywords:** patient satisfaction, validation studies, service quality, hospitals, public-private partnership, Peru

## Abstract

During recent years, public–private partnerships (PPPs) in the health sector have been an attractive alternative for improving healthcare services in developing countries such as Peru. Therefore, it is fundamental to consider a comprehensive set of healthcare qualities, like the HEALTHQUAL scale, when we measure dimensions of healthcare service quality. Currently, no studies have applied HEALTHQUAL in Peruvian hospitals. The purposes of this study were to (1) validate and evaluate the application of the HEALTHQUAL scale to measure user satisfaction in outpatient services at two PPP hospitals in Peru; and (2) test the relationship between user satisfaction, efficiency, and loyalty. A descriptive, cross-sectional study based on the HEALTHQUAL scale was carried out at the end of 2018. The measurement items were satisfaction with healthcare personnel, satisfaction with nonhealthcare personnel, satisfaction with facilities and equipment, perception of efficiency, and trust. The scale was administered to a nonprobability sample of 250 users who attended one of two PPP hospitals—Barton and Kaelin. The application of partial least squares path modeling significantly impacted on the perceived efficiency in the items of healthcare personnel, nonhealthcare personnel, and facilities and equipment. The HEALTQUAL scale demonstrated sufficient validity and thus can be applied for measuring user satisfaction in PPP hospitals.

## 1. Introduction

### 1.1. Quality of the Services in Hospital Management

Current efforts to improve the delivery of healthcare services globally have highlighted quality as a fundamental and intrinsic value of health organizations worldwide [1,2]. Quality considers the user’s perspective and satisfaction with the service received, with the user determining its level in a framework of continuous improvement. This framework should include objective and measurable characteristics in any organization [3,4,5].

Applied to the health sector, this concept is related to the application of technology and science to maximize the benefits and reduce the risks in the healthcare process, expecting a positive impact on health indicators, an improvement in health problems, and the satisfaction of patients and families [6,7]. According to Avedis Donabedian [8], the quality of healthcare is defined as “the degree to which the most desirable means are used to achieve the greatest possible improvement in health. However, as the consequences of care are manifested in a future that is often difficult to know, what is important is the outcome expectations that could be attributed to health care in the present.”

For this reason, the implementation of quality management strategies represents a key factor for achieving success in healthcare organizations [9,10]. One of these tools is the management of user perception of the service received, since the performance of organizations is closely linked to the intention of using a service again in the future, which is a result of user satisfaction [11,12].

In this context, user satisfaction is an indicator of the quality of the service [13], resulting in a value judgment as a consequence of the interaction of the user with the service, and is related to various factors such as care, empathy, credibility, and responsiveness [14,15].

For years, studies have been suggesting that the quality of a service is one of the most important determinants of customer satisfaction and trustworthiness [16,17,18]. Therefore, the objective of all organizations is to permanently improve the quality of the services provided and thus to satisfy and retain their clients. For this, managers need to continuously analyze the context and drivers of the quality of service provided by their organizations, the customer satisfaction, and the value perceived by the users of their services. In particular, the central question to be investigated by managers is how all of these above-mentioned factors or variables relate to each other to understand and explain the future behavior of their clients [19].

### 1.2. Public–Private Associations (PPPs) as an Organization Model in Health

Public–private partnerships (PPPs) have emerged in recent years as a strategy to expand coverage and to improve healthcare services in different Latin American countries, proving to be an effective model to fill the gap in infrastructure and services, as well as to improve the quality of the existing infrastructure [20]. PPPs are defined as long-term contracts between a private party and a public entity, which provide a public good or service in which the private party assumes a significant risk and the responsibility for management and has performance indicators [21]. Similarly, the Organization for Economic Cooperation and Development [22] defines PPPs as agreements between the government and one or more private partners, under which the latter provide a service in such a way that the government’s service delivery objectives are aligned with the objectives of obtaining profit from the private sector, and where effectiveness depends on an adequate transfer of risk from the private sector.

As both of the above-mentioned documents regarding PPPs highlight, although there is no single and generalized definition of these schemes in different countries, PPP models facilitate broad and participatory solutions between the public and private sectors for the provision of goods and public services in an integral way. There are usually two mechanisms by which to pay the private sector for the responsibilities—(a) through government payments to the private sector according to the degree of quality and the results of the services provided to users, defined contractually; or (b) through direct payments from the users who receive these services and are supplemented by subsidies paid by the government [21].

The need for infrastructure and quality healthcare services in developing countries has highlighted the PPP model as a very useful alternative for solving the financial restrictions and management capacity faced by the governments, expanding the coverage of their healthcare systems and improving the quality of the services provided. In this sense, three main benefits of PPPs in the health sector have been recognized—(i) efficiency improvement, (ii) improvements in the quality of services, and (iii) improvements in the allocation of risks and resources [23,24]. Evidence from Spain, Chile, England, and Germany [25,26,27,28] have described the role of PPPs in optimizing resources and improving productivity and efficiency, given that PPPs usually assure greater quality and quicker adaptation to market needs compared to public management processes.

In Peru, the first two hospitals operating under the PPP model belong to the EsSalud (Peruvian social security health system), namely, the Alberto L. Barton Thompson Hospital in the Callao district (started in 2009) and the Guillermo Kaelin de la Fuente Hospital in the Villa María del Triunfo district, both located in the Region of Lima (Peru). Both hospitals have been operating for 11 years, but there are no published studies that describe the experience of PPPs and their relationship with quality and user satisfaction. To date, there is also no valid scale to evaluate user satisfaction in PPP hospitals in Peru.

Therefore, given the scarcity of research in Latin America, and particularly in Peru, on scales to measure the quality of service perceived by healthcare users (for example, the patient, the patient’s relatives, or the general public) in a hospital setting, our work focuses on validating an expanded version of a healthcare service quality scale, namely HEALTHQUAL, based on the previous work of Lee and Kim [29] and Miranda et al. [9], which consists of five constructs, and which can be further adapted to other Latin American countries. In addition, our study proposes a seven-hypothesis research model to analyze the cause-effect relationships between these five constructs.

### 1.3. The HEALTHQUAL Scale, Dimensions, and Items

For years, SERVQUAL has been used as a multidimensional scale, with high validity and reliability, to assess the quality in health-related organizations and other sectors [30]. Moreover, adaptations of the SERVQUAL scale in the health field has allowed evaluation of the perceptions of patients on the quality of care and services in healthcare facilities [31,32].

Since Myers [33] first introduced the concept of the quality of healthcare services, it has been measured using various dimensions [34,35,36] that have evolved according to the research agenda [37].

Managing quality in healthcare services requires an efficient and integral approach for collecting information about user care. In this sense, HEALTHQUAL is a multidimensional scale to measure the quality of services in modern medical care that evaluates items from the patient’s perspective. The set of items considers dimensions such as improvement in care services, quality, efficiency, safety, and empathy and loyalty of healthcare users. Although various existing scales include most of the items suggested by previous studies [9,29,34,35,36,37], HEALTHQUAL may require modifications or changes over time; thus, by continuous improvement, it can more precisely measure the quality of medical care provided to patients, considering different geographical areas, local cultures, and other factors [29]. Therefore, although adaptations of the HEALTHQUAL survey are frequently used to measure perceived satisfaction, to date, no studies have been conducted using the HEALTHQUAL scale in PPP hospitals in Peru.

Studies using the HEALTHQUAL scale usually include four components, namely, healthcare personnel, efficiency measures, nonhealthcare personnel, and physical facilities [9], or include five components—empathy, tangibles, safety, efficiency, and improvements of care service [37].

In our study, the quality of service was analyzed using a version of HEALTHQUAL adapted from the previous work of Lee and Kim [29] (i.e., five dimensions and 32 items) and Miranda et al. [9] (i.e., four dimensions and 25 items), considering the healthcare personnel and nonhealthcare personnel, the facilities and equipment of the healthcare center, and the perceived efficiency and the loyalty or intention to reuse the service. Thus, our HEALTHQUAL scale consists of five constructs and a total of 34 items—(1) satisfaction with healthcare personnel (10 items); (2) satisfaction with nonhealthcare personnel (4 items); (3) satisfaction with facilities and equipment (6 items); (4) perceived efficiency (11 items); and (5) loyalty (3 items). This last component of the scale—loyalty or intention to reuse the service—reflects the overall evaluation of the quality of service perceived by the user, and is related in some way to the empathy component of Lee and Kim’s work [29] and the global satisfaction toward the healthcare service.

An initial pretest was carried out on the HEALTHQUAL scale before the data collection phase to ensure that it was adjusted to the profile of healthcare users in Peru. Participants were both academic and healthcare professionals. Decisions to adapt the questionnaire were based on two criteria—(a) the suitability of the questions for the healthcare services, and (b) the ability of the patients to answer the questions without feeling confused, doubtful, or insecure.

An initial scale composed of 38 items was then administered to a small sample of patients (i.e., healthcare users) as a pretest to obtain more information and to refine the items. The results showed that the respondents perceived that some items were somewhat redundant. Since this redundancy caused confusion, we decided to remove four items from the measurement scale. Therefore, our final scale included 34 items that represent the five dimensions of the quality of service perceived by users of healthcare services.

### 1.4. Research Model and Hypothesis

#### 1.4.1. Healthcare Personnel Dimension (HP)

Healthcare personnel represent the most important component of healthcare services and include different groups and professions in a healthcare organization. From the point of view of satisfaction with the quality of services, it is very important that healthcare professionals keep proper interpersonal relationships with patients in a dynamic, continuous, and timely manner. In this context, the perception of patient satisfaction depends almost entirely on the care received from healthcare personnel [38]. Therefore, we propose the following hypotheses:

H1: The better the performance of healthcare personnel, the greater the efficiency in healthcare; and
H2: The better the performance of healthcare personnel, the greater loyalty of users to the healthcare service.

#### 1.4.2. Nonhealthcare Personnel Dimension (NHP)

In the context of the healthcare system, there are different factors that intervene directly or indirectly with patient satisfaction. One of them is the group of nonhealthcare professionals, involving administrative personnel who work in auxiliary services or that complement medical care. Several studies have related the care provided by the administrative services to the quality of care perceived by users who attend healthcare services [39]. Therefore, we propose the following hypotheses:

H3: The better the performance of nonhealthcare personnel, the greater the efficiency in healthcare; and
H4: The better the performance of nonhealthcare personnel, the greater loyalty of the user to the healthcare services.

#### 1.4.3. Facilities, Equipment, and Tangible Dimension (FET)

The physical space, infrastructure, equipment, and materials are essential to provide proper conditions to carry out quality medical care [40]. Based on the above, we propose the following hypotheses:

H5: The better the conditions of the facilities, equipment, and tangibles, the greater the efficiency in healthcare; and
H6: The better the conditions of the facilities, equipment, and tangibles, the greater the loyalty of the user to the healthcare service.

#### 1.4.4. Relationship Between Efficiency (EFI) and Loyalty (L)

The measurement of efficiency in a hospital setting is related to the efficient use of resources and the management of process waiting times. Considering that, in many of the health sectors, resources are deficient and scarce, efficiency refers the adequate use of resources in order to maximize benefits for the user and the healthcare services. Likewise, at the hospital management level, efficiency analysis allows one to improve the use of resources and the reorganization of services to add value for healthcare users [41,42].

User loyalty is the result of quality care and represents its continuity through a direct relationship where satisfied customers become loyal customers, committed to the organization and willing to repeat the experience and share it [43]. Based on the above, we propose the following hypothesis:

H7: The greater the efficiency of the healthcare service provided, the greater the patient’s loyalty to that healthcare service.

**Figure 1:**
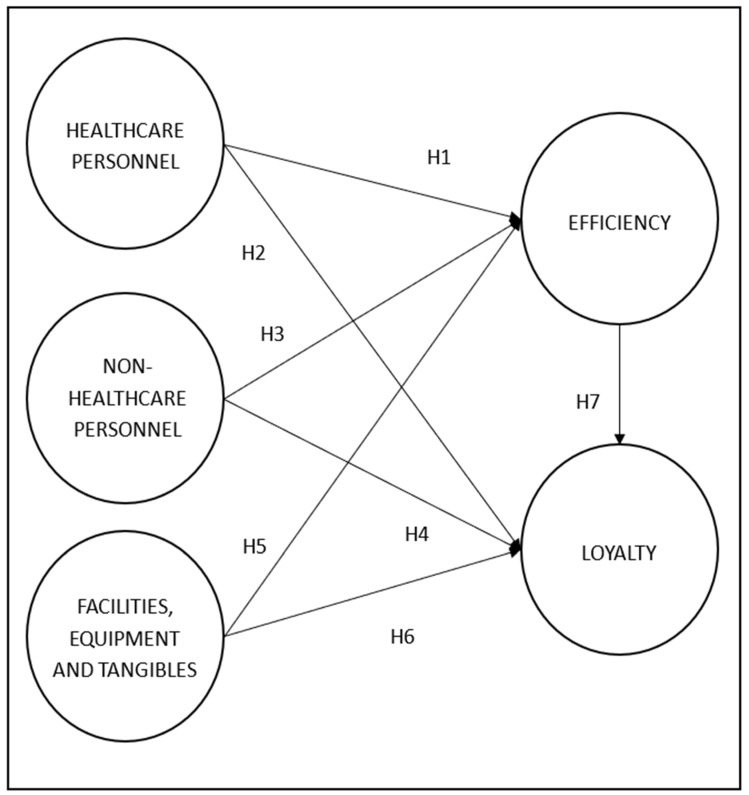
Model of the hypotheses.

The above hypotheses are summarized in Figure 1.

H1: Healthcare personnel positively impact efficiency;
H2: Healthcare personnel positively impact loyalty;
H3: Nonhealthcare personnel positively impact efficiency;
H4: Nonhealthcare personnel positively impact loyalty;
H5: Facilities, equipment, and tangibles positively impact efficiency;
H6: Facilities, equipment and tangibles positively impact loyalty; and
H7: Efficiency positively impacts loyalty.

## 2. Materials and Methods

A descriptive, exploratory, cross-sectional study was carried out during 2018. Applying nonprobability sampling, the HEALTHQUAL scale was applied to a sample of 250 users who attended one of the two PPP hospitals in Lima, Peru (Alberto L. Barton Thompson Hospital and Guillermo Kaelin de la Fuente Hospital), which serve an average of 250,000 insured patients annually.

The study consisted of three phases—(I) scale development, (II) evaluation of the validity and reliability, and (III) scale evaluation.

### 2.1. Measurement Instrument

A questionnaire consisting of 34 items representing the five dimensions of the quality of service perceived by users of healthcare services was used (see Table 3). All items were measured on a 7-point Likert scale, where 7 was “totally agree” and 1 was “strongly disagree”. The questions used were based on the HEALTHQUAL scale described above. This HEALTHQUAL scale measures the constructs of healthcare personnel, nonhealthcare personnel, facilities and equipment, and efficiency. For the purposes of this study, the “loyalty” construct was added in order to validate a new construct that measures variables related to the continuity of care after the provision of the healthcare service. The indicators of this construct were adapted from various studies that used a loyalty scale [44,45,46,47,48].

### 2.2. Variables

Efficiency and loyalty were established as dependent variables, and healthcare personnel, nonhealthcare personnel, and facilities and equipment were used as independent variables, as shown in Figure 1.

### 2.3. Application of the Questionnaire

The questionnaire was distributed according to sex and age of the participants, in keeping with the distribution of the population at both PPP hospitals. Previously, we asked permission from the PPP managers at both hospitals for the application of the surveys, and the external interviewers were trained in the HEALTHQUAL methodology. The data collection was anonymous, and we protected the confidentiality of the database.

### 2.4. Data Processing and Analysis

An Excel spreadsheet was generated with the information obtained from the surveys, and analysis was subsequently performed using the partial least squares (PLS) methodology to validate the model and the hypotheses proposed. This statistical methodology is based on an iterative combination of principal components analysis and regression analysis. This process corresponds to a model developed by Wold to reflect the conditions present in social and behavioral sciences [49].

The evaluation of a model using the PLS methodology has three phases—(a) evaluation of the global model, (b) evaluation of the measurement model, and (c) evaluation of the structural model.

The global model was analyzed by measuring the standardized root mean squared residual (SRMR). A value below 0.08 indicates that the PLS path model provides a sufficient fit between the proposed model and the data, as described by Hu and Bentler [50].

For the evaluation of the measurement model, factor loadings greater than 0.707 suggest the individual viability of the indicators for each construct [51].

Discriminant validity analysis was performed using the Heterotrait–Monotrait Ratio (HTMT) criterion, which assesses the average of the Heterotrait–Heteromethod correlations of the variables estimated in Mode A [52]. The HTMT level must be below at least the 0.90 value to conclude that the constructs reach validity [53]. Likewise, discriminant validity indicates to what extent a given construct is different from the other constructs [54]. As for the evaluation of the structural model, the problems of collinearity were analyzed according to the variance inflation factor (VIF) values.

## 3. Results

A total of 250 patients who attended either one of the PPP hospital complexes were surveyed. The age range was from 35 to 54 years, and 128 respondents were women, while the other 122 were men (Table 1).

### 3.1. Global Model

Based on the results, the Standarized Root Mean-Square (SRMR) was equal to 0.060, which means that the model fits the data (Table 2).

### 3.2. Measurement Model

As shown in Table 3, in reference to the individual viability of each item, the factor loadings obtained in most of the indicators were greater than 0.707, demonstrating that the proposed indicators are suitable for the constructs.

Table 4 shows that, according to the HTMT criterion, two values are less than 0.85 and one is less than 0.90. Therefore, all three constructs are valid. The five constructs are also shown to have discriminant validity, making them different from each other in their ability to measure satisfaction.

### 3.3. Structural Model

Following the direction of Hair et al. [55], the problems of collinearity between the constructs are ruled out, since the VIF values are less than five (see Table 5).

Regarding the structural model, as shown in Table 6, the hypotheses that nonhealthcare personnel (H4) and facilities and equipment positively impact loyalty to the service were not statistically significant. The remaining five were statistically significant, with *p* < 0.005.

The effects on the endogenous variables are represented in Table 7. According to the criteria of Hair et al. [55], the EFI and L constructs have a moderate explanatory power (R^2^ > 0.5).

The predictive relevance of the constructs according to the Stone–Geisser test (Q^2^) reached positive values.

The evaluation of the size of the effects (f^2^) shows various results—(a) a small effect (0.02 ≤ f^2^ < 0.15) in H2, H3, and H1; and (b) a moderate effect (0.15 ≤ f^2^ < 0.35) in H5 and H7. Likewise, it should be noted that the effect size of hypotheses H4 and H6 is below the lower limit of the small effect (0.02).

## 4. Discussion

This study used the PLS methodology and was carried out in three phases. The evaluation of the global model showed that the model fits the data. Likewise, the evaluation of the measurement model showed that the proposed indicators are adapted to the constructs of “healthcare personnel,” “nonhealthcare personnel,” “facilities and equipment,” “efficiency,” and “loyalty.” Furthermore, these five constructs have discriminant validity, making them different from each other in their ability to measure the satisfaction of the users of healthcare services in Peru. Regarding the evaluation of the structural model, the dependent characteristics of healthcare personnel, nonhealthcare personnel, and the facilities and equipment that the hospital has all positively impact efficiency. However, only the characteristics dependent on healthcare personnel and efficiency are associated with the loyalty that the user develops toward the healthcare services.

Regarding the evaluation of reliability and validity of the instrument, we found that the HEALTHQUAL scale presented adequate psychometric estimates, since the constructs have discriminant validity and the factor loadings showed that the proposed indicators are adequate for the constructs. These results are consistent with the study carried out by Lee and Kim [37], where the use of the HEALTHQUAL scale demonstrated validity and reliability to examine the quality dimensions of a healthcare service in South Korea based on five components—namely, empathy, tangibles, safety, efficiency, and the degree of improvement of the care service.

On the other hand, the association found between efficiency and the factors related to healthcare personnel, nonhealthcare personnel, facilities and equipment is similar to the study reported by Jiménez [56], who, in a conceptual review, stated that medical care is a complex process where the patient’s interaction with the healthcare team, in addition to technology and infrastructure, plays a relevant role in achieving quality care and service efficiency. In this sense, it is important to highlight that the PPP model in healthcare has arisen due to noncompliance with the minimum quality standards by public healthcare centers, and therefore, PPPs represent an excellent mechanism by merging the advantages of the regulations of the public sector and the operations of the private sector. In this sense, PPPs offer healthcare services with good infrastructure, modern equipment, and trained healthcare personnel, and they promote a quality culture based on productivity and efficiency [57].

In Peru, EsSalud was the first institution to promote investment projects in the health sector through a PPP model that included white coat services, where the operator, in addition to promoting infrastructure and equipment, includes assistance and administrative services. This is a unique experience that exceeds what has been done so far in Latin America [58]. The model has shown improvements in the quality of services through obtaining certifications such as ISO 9001-2015 for central sterilization, hemodialysis services, and operation of the supply chain to the end consumer at the Alberto Leonardo Barton Thompson and Guillermo Kaelin de la Fuente hospitals, both in the region of Lima (Peru) [59].

Regarding efficiency, both hospitals have reduced waiting times, have implemented a “zero paper” management process, and have a “cost per insured user” that is less than other hospitals, showing user satisfaction indicators over 80% [60].

Another of the findings of this study shows that the “healthcare personnel” factor positively impacts loyalty, which is related to that which was previously mentioned regarding the white coat services operated by PPPs, where healthcare personnel (i.e., doctors, nurses, and technicians) and administrative staff (i.e., nonhealthcare personnel) are continuously selected and trained to provide preventive healthcare services and good quality. All of these factors have a positive impact on user satisfaction, improving their perception of the service and the intention of using it again. The association between efficiency and loyalty obtained in this study highlights that if an efficient service is provided, the patient’s loyalty increases. However, to our knowledge, there are no studies in Latin America that have explained this relationship in the health sector; therefore, this is a pioneering study and contributes to closing the existing gap in the literature on the topic.

In our study, the design of a new loyalty construct incorporated indicators to determine if the patient would return to the hospital again, if the patient recommended the hospital to family and friends, and if the user would repeat the healthcare experience due to the effectiveness of the service received. We could not find a study in Latin America that included this construct of loyalty in the tools to measure the quality of healthcare services; thus, it would be relevant if it can be taken into account for future evaluations or research along these lines.

On the other hand, the adaptation of the SERVQUAL scale, called HEALTHQUAL, carried out in Spanish hospitals by Mirada et al. [44], involved a validation of the new construct and an evaluation of its reliability. However, there is no evidence in Peru of its validity being applied in public healthcare services or private hospitals. In this sense, this study is the first in Latin America to validate the use of the HEALTHQUAL scale to measure user satisfaction in PPP hospitals using the PLS structural equation modeling methodology. Instead, various studies on user satisfaction in healthcare services carried out in Latin America [61,62,63] have used other types of analysis, such as bivariate and multivariate analyses, the Student’s *t*-test, and analysis of variance (ANOVA). These previous studies show various interpretations, where the overall user satisfaction is analyzed in a very general and ambiguous way, which does not allow one to establish proposals for solutions to problems with precision. Furthermore, some of these studies only sought to validate the instrument, while the present study validates both the theoretical model (i.e., the hypotheses) and the measurement instrument.

Finally, this research has some limitations. First, there may be a potential selection bias, because the sampling was nonprobabilistic; therefore, these findings cannot be generalized to the entire population. Second, the study has an information bias, due to the fact that it was not possible to evaluate other characteristics that really measure the level of satisfaction of external users, which can lead to imprecise estimates or a poor classification in interest groups. Finally, due to the cross-sectional design and the absence of temporality, it is not possible to attribute strong causality between the evaluated characteristics. Future research might consider a longitudinal design that at least contemplates information at two different moments in time.

## 5. Conclusions

In conclusion, our research showed that the HEALTHQUAL scale presents optimal psychometric properties related to content and construct validity, which suggests that it is a valid and applicable instrument to measure the level of user satisfaction at PPP hospitals. On the other hand, our research showed that the constructs healthcare personnel, nonhealthcare personnel, and facilities and equipment significantly impact perceived efficiency. However, only the characteristics dependent on healthcare personnel are associated with a user’s loyalty to the healthcare services. The relationship between each of the components or variables of the quality of the healthcare service that affect patient satisfaction and the level of satisfaction of these with the healthcare centers in the PPP model were very high and positive in Peru.

## Figures and Tables

**Table 1:** Distribution of the surveyed patients according to age and gender in the two hospital complexes.

Age	Hospital 1 ^1^		Hospital 2 ^2^		Total
	Male	Female	Male	Female	
18–34	16	16	17	18	67
35–54	25	24	25	27	101
55–74	16	18	12	14	60
+75	6	7	5	4	22
Total	128		122		250

^1^ Alberto L. Barton Thompson Hospital; ^2^ Guillermo Kaelin de la Fuente. Both hospitals are located in the Region of Lima (Peru).

**Table 2:** The global model.

Indicator	
SRMR ^1^	
Saturated Model	0.0643
Estimated Model	0.0643

^1^ Standardized root mean squared residual.

**Table 3:** Results of the measurement model.

Construct/Indicator		Factor Loadings	Composite Reliability	AVE ^1^	ρA ^2^
Healthcare Personnel (HP)			0.952	0.667	0.947
HP1	The professionalism of the healthcare personnel is very high	0.7574 ***			
HP2	The kindness and courtesy of the healthcare personnel is very high	0.7497 ***			
HP3	Confidence in the healthcare personnel is very high	0.8000 ***			
HP 4	The healthcare personnel provide a highly personalized service	0.7828 ***			
HP5	Communication with the healthcare personnel is very good	0.8387 ***			
HP6	Individualized care of the healthcare personnel to the problems of the patient is very good	0.8623 ***			
HP7	The interest of the healthcare personnel in attending to the patient’s problems is very high	0.8733 ***			
HP8	The concern of the healthcare personnel to understand the problems of the patient is very high	0.8514 ***			
HP9	The prestige of the medical staff is very good	0.8554 ***			
HP10	Doctors explain in detail the diagnoses and treatment of a disease	0.7858 ***			
Nonhealthcare personnel (NHP)			0.957	0.848	0.941
NHP1	The professionalism of the non-healthcare personnel is very high	0.9034 ***			
NHP2	The kindness and courtesy of the nonhealthcare personnel is very good	0.9250 ***			
NHP3	Individualized care of the nonhealthcare personnel to the problems of the patient is very good	0.9335 ***			
NHP4	The interest of the nonhealthcare personnel in attending to the patient’s problems is very high	0.9221 ***			
Facilities, equipment, and tangibles (FET)			0.894	0.585	0.865
FET1	The cleanliness of the facilities is very high	0.6434 ***			
FET2	The equipment of the healthcare center is very good	0.7434 ***			
FET3	The accessibility of the healthcare center is very good	0.8034 ***			
FET4	The appearance and presence of the healthcare personnel is very good	0.8435 ***			
FET5	The appearance and presence of the non-healthcare personnel is very good	0.8051 ***			
FET6	There is clear signage for each department in the hospital	0.7335 ***			
Efficiency (EFI)			0.919	0.507	0.906
EFI1	The hospital provides many facilities to arrange a medical appointment	0.7402 ***			
EFI2	The level of bureaucracy is minimal (very low)	0.7282 ***			
EFI3	The waiting time in the healthcare center before entering a medical consultation is adequate	0.6685 ***			
EFI4	The hospital provides a very good computerized service	0.6844 ***			
EFI5	The speed of ancillary testing is very high	0.7036 ***			
EFI6	Complaint resolution is very fast and satisfactory	0.7796 ***			
EFI7	The time spent focusing on the care of each patient is adequate	0.7330 ***			
EFI8	The hours of the healthcare center are very wide and adequate	0.6492 ***			
EFI9	Medical expenses are reasonable	0.6465 ***			
EFI10	The existence of improvement in the medical condition as a result of the efforts and treatment by medical personnel is very high	0.7512 ***			
EFI11	The occurrence of side effects when patients take their medicines is very low	0.7382 ***			
Loyalty (L)			0.945	0.852	0.913
L1	The patient is treated again in this hospital	0.9169 ***			
L2	The patient recommends this hospital to his friends and family	0.9432 ***			
L3	Visiting the hospital again for its effectiveness in organization and service	0.9081 ***			

^1^ Average Variance Extracted. ^2^ Dijkstra-Henseler’s rho. *** *p* < 0.001; based on a bootstrapping of 10,000 samples with a two-tailed test.

**Table 4:** The measurement model—discriminant validity.

Heterotrait–Monotrait Ratio (HTMT)					
	HP	NHP	FET	EFI	L
HP					
NHP	0.63				
FET	0.679	0.593			
EFI	0.73	0.661	0.756		
L	0.697	0.588	0.626	0.785	
Fornell–Larcker Criterion					
	HP	NHP	FET	EFI	L
HP	0.817				
NHP	0.613	0.921			
FET	0.672	0.531	0.765		
EFI	0.682	0.613	0.672	0.712	
L	0.65	0.546	0.566	0.72	0.923

**Table 5:** The variance inflation factor (VIF) values of the structural model.

Construct	HP	NHP	FET	EFI	L
HP				1.907	2.184
NHP				1.655	1.792
FET				1.726	2.02
EFI					2.504
L					

**Table 6:** The structural model.

Hypothesis	Suggested Effect	Path Coefficient	Confidence Interval 99%	Student’s *t*	*p*-Value
H1: HP -> EFI	+	0.3331 ***	[0.2410; 0.4223]	6.0374	0.0000
H2: HP -> L	+	0.2525 ***	[0.1039; 0.4118]	2.7147	0.0033
H3: NHP -> EFI	+	0.2338 ***	[0.1547; 0.3252]	4.4977	0.0000
H4: NHP -> L	+	0.0856	[−0.0566; 0.2069]	1.0763	0.1409
H5: FET -> EFI	+	0.3428 ***	[0.2545; 0.4314]	6.3340	0.0000
H6: FET -> L	+	0.0595	[−0.0555; 0.1763]	0.8476	0.1983
H7: EFI -> L	+	0.4550 ***	[0.3345; 0.5904]	5.9320	0.0000

*** *p* < 0.001; based on a bootstrapping of 10,000 samples with a one-tailed test.

**Table 7:** Effects on the endogenous variables.

Construct	R^2^	Q^2^	f^2^	Direct Effect	Correlation	Variance Explained
EFI	0.6007	0.2792				
H1: HP			0.1457	0.3331	0.682	22.70%
H3: NHP			0.0827	0.2338	0.6131	14.30%
H5: FET			0.1705	0.3428	0.6715	23.00%
L	0.572	0.4529				
H2: HP			0.0682	0.2525	0.6501	16.40%
H4: NHP			0.0096	0.0856	0.5457	4.70%
H6: FET			0.0041	0.0595	0.5656	3.40%
H7: EFI			0.1932	0.455	0.7197	32.70%
